# Supplementary figures and images for: Endomitosis controls tissue-specific gene expression during development
Source: PLoS Biol. 2022 May 24;20(5):e3001597. doi: 10.1371/journal.pbio.3001597 (PMC9129049; doi:10.1371/journal.pbio.3001597)

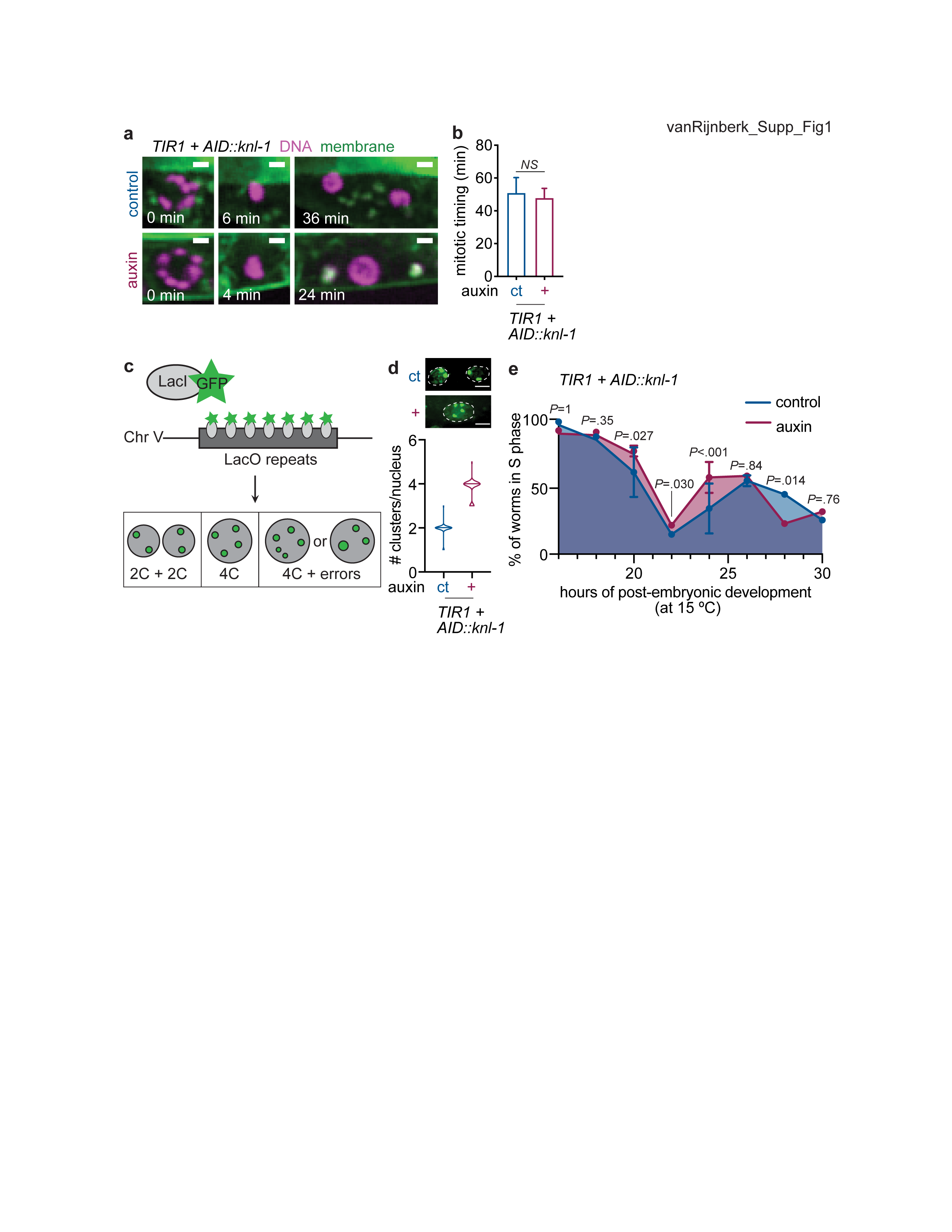

Supplement: S1 Fig — (A) Stills of time-lapse videos of cells undergoing endomitosis in the absence (control) or presence of auxin, showing intestinal H2B-mCherry (DNA, shown in magenta) and GFP-PH (membrane, shown in green). Scale bar is 2 μm. Time stamp indicates time after nuclear envelope breakdown. (B) Quantification of mitotic timing, the duration from nuclear envelope breakdown to nuclear envelope reformation, in the absence (ct, n = 4) or presence (+, n = 7) of auxin. Bar graph showing mean and error bars indicate SEM. P values were calculated by unpaired Student t test. (C) Overview of LacI/LacO system for detection of individual chromosomes in polyploid cells. A series of LacO repeats present on chromosome V are visualized upon heat shock–induced expression of a LacI fused to GFP. After endomitosis in L1, a binucleated cell with two 2C nuclei or a mononucleated cell with a single 4C nucleus should show 4 individual chromosomes if no segregation errors occurred. If errors did occur during segregation, an alternate number of individual chromosomes should be visible. (D) Fluorescent images and violin plots showing chromosome cluster counts of fluorescent LacI::GFP foci in control (ct, n = 98) and auxin-treated (+, n = 82) worms. To distinguish LacI::GFP signal from cytoplasmic autofluorescent vesicles, only nuclear dots were counted as chromosome clusters. Error bars represent min and max values, and horizontal bars represent median. Scale bar is 5 μm. (E) Average percentage of animals in which intestinal cells are undergoing G2 or S phase, determined by the presence of CYB-1DB::mCherry, during endomitotic and subsequent endoreplicative cycles in the first larval stage for 60 to 200 worms per condition per time point, in 3 replicate experiments. x Axis depicts hours after starved L1 animals were placed on food at 15 degrees and starts at 16 hours. P values were calculated by Fisher exact. Error bars represent standard deviation between replicate experiments, if applicable. Underl [file pbio.3001597.s001.tif]

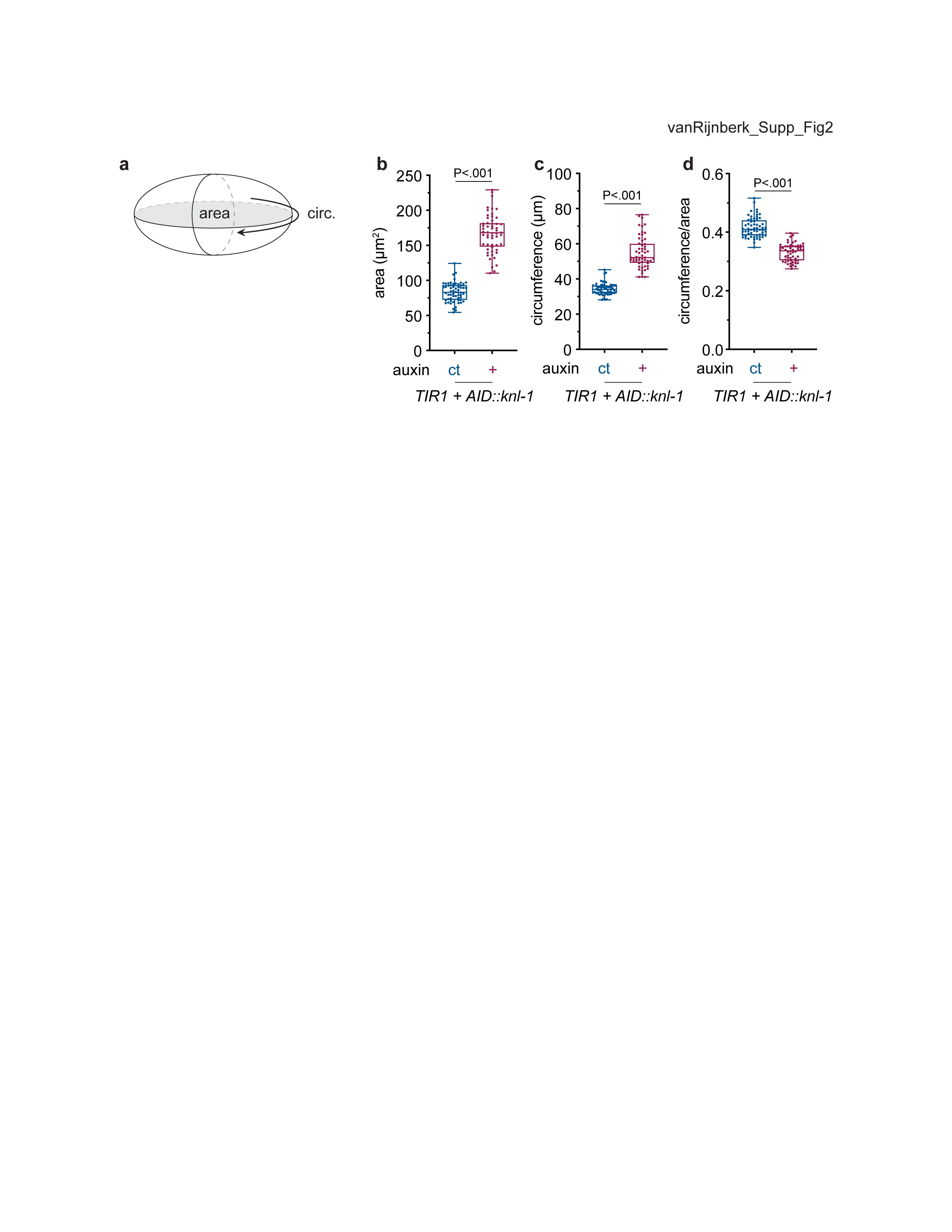

Supplement: S2 Fig — (A) Schematic depicting the nuclear parameters measured, area and circumference of a nuclear midplane sections. (B–D) Boxplots depicting nuclear section area (B), circumference (C) and nuclear circumference-to-area ratio (D) in binucleated (ct, n = 60) or mononucleated (+, n = 56) cells. Measurements were made at the midplane of the nucleus. Boxplots indicate the median and 25th to 75th percentile, error bars indicate min to max values, and individual values are shown as dots. P values were calculated by Mann–Whitney (B, C) and unpaired Student t test (D). Underlying data can be found in S1 Data. (TIF) [file pbio.3001597.s002.tif]

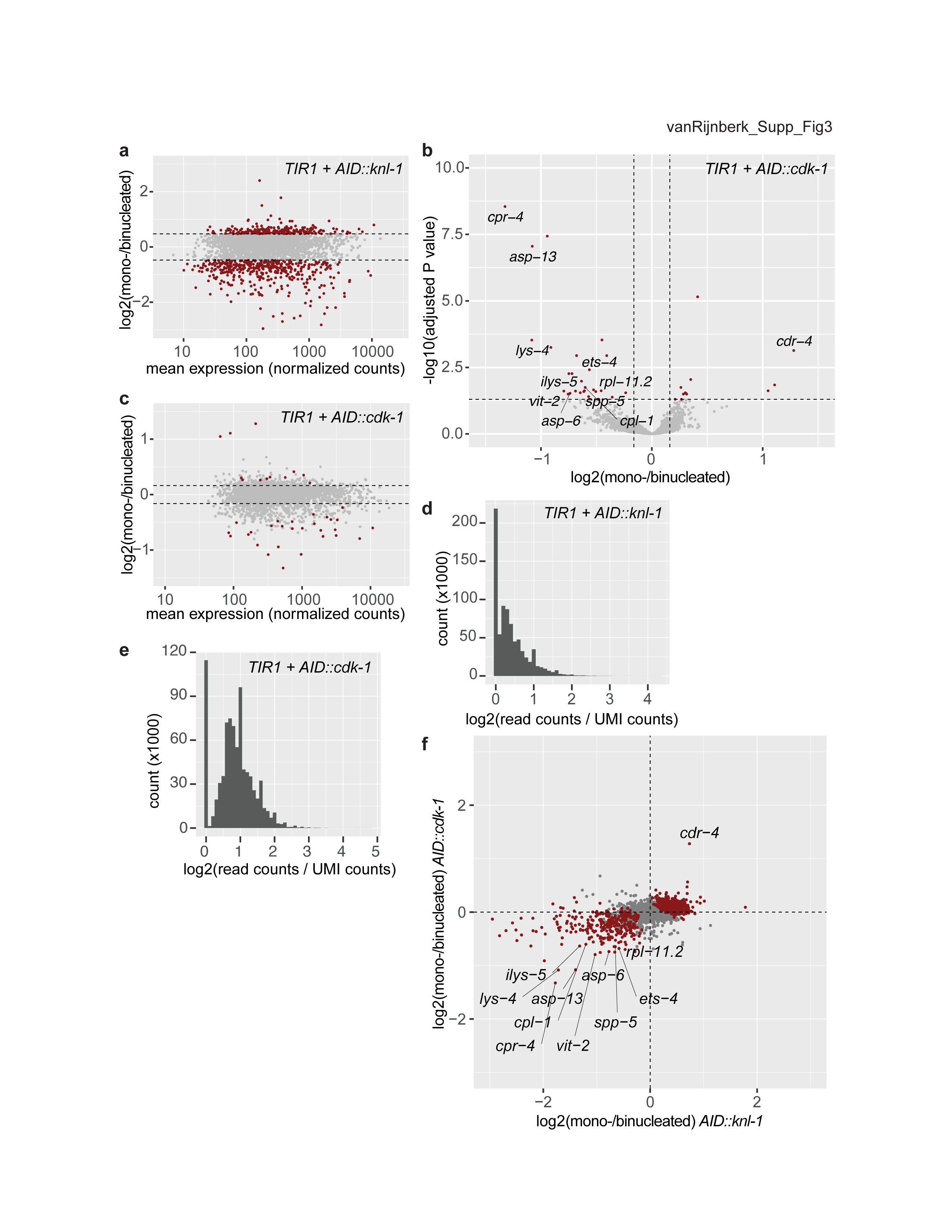

Supplement: S3 Fig — (A–C) Volcano and MA plots of RNA sequencing data depicting the transcriptional gene up- and down-regulation in worms with a mononucleated intestine, compared to worms with a binucleated (wild type) intestine in animals containing either AID::knl-1 (A) or AID::cdk-1 (B, C), in relation to gene expression levels. Red dots represent genes that are differentially expressed with an adjusted P value below 0.05 and belong to the top 25% regarding absolute log2(foldchange). (D, E) Histogram depicting the log2 of read counts per unique molecular identifier (UMI) for each gene found in animals containing either AID::knl-1 (D) or AID::cdk-1 (E). (F) Dot plot depicting the correlation between differential expression in AID::knl-1 (x-axis) and AID::cdk-1 (y-axis) animals with a mononucleated versus binucleated intestine. Red dots represent genes that are differentially expressed in the combined dataset with an adjusted P value below 0.05. Genes significantly differentially expressed in both AID::knl-1 and AID::cdk-1 comparisons individually were annotated with their gene name (excluding genes without a gene name). Underlying data are available at the Gene Expression Omnibus, identifier GSE169330, and in S1–S3 Data. AID, auxin-inducible degron. (TIF) [file pbio.3001597.s003.tif]

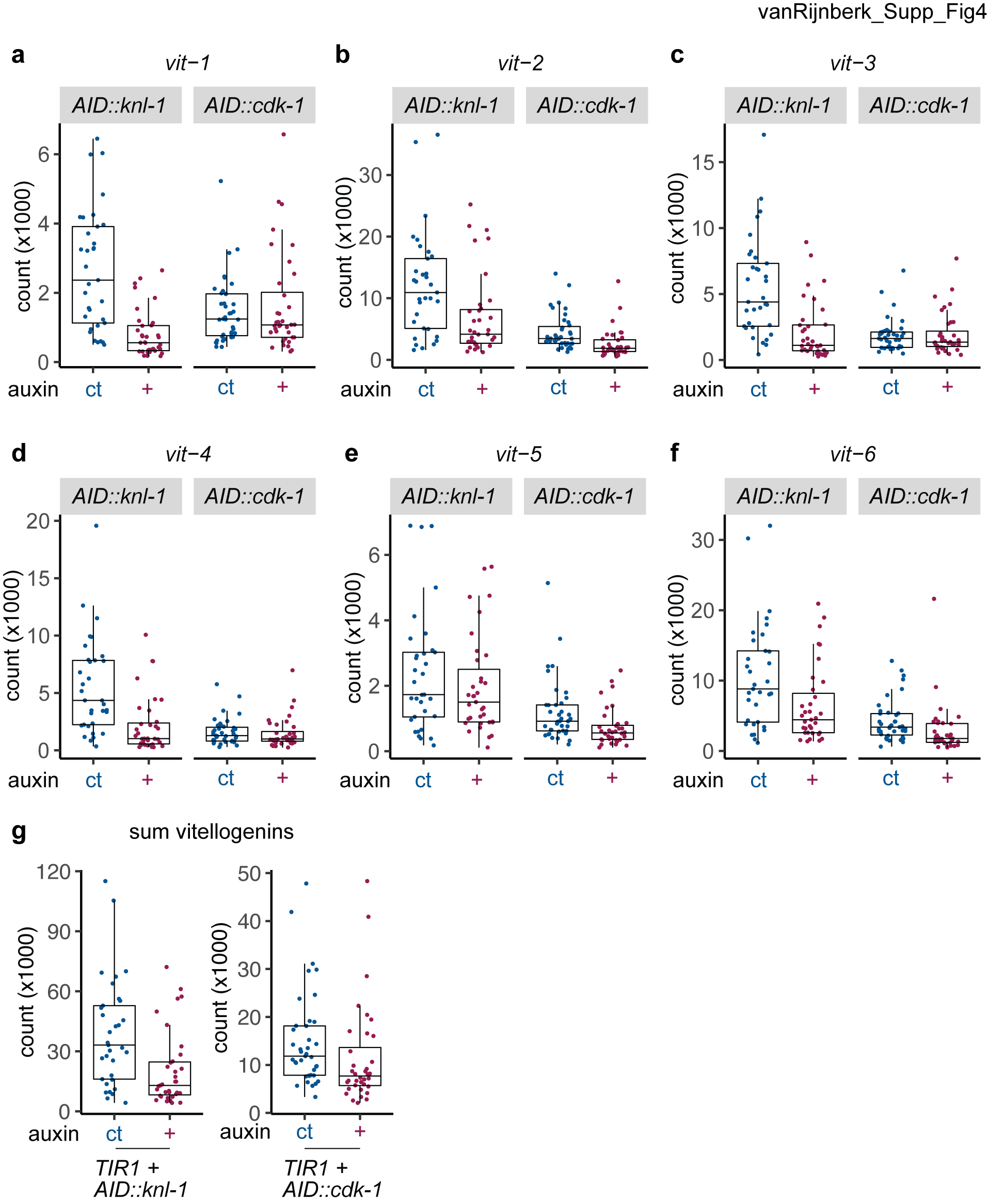

Supplement: S4 Fig — (A–G) Tukey boxplots showing the separate (A–F) and accumulated (G) RNA expression levels for all 6 vitellogenin genes (vit-1 through vit-6) in auxin-control (ct, n = 33 for AID::knl-1 and n = 39 for AID::cdk-1) or auxin-treated (+, n = 35 for AID::knl-1 and n = 36 for AID::cdk-1). Each dot represents the expression in one worm. Underlying data are available at the Gene Expression Omnibus, identifier GSE169330, and in S1 Data. AID, auxin-inducible degron. (TIF) [file pbio.3001597.s004.tif]

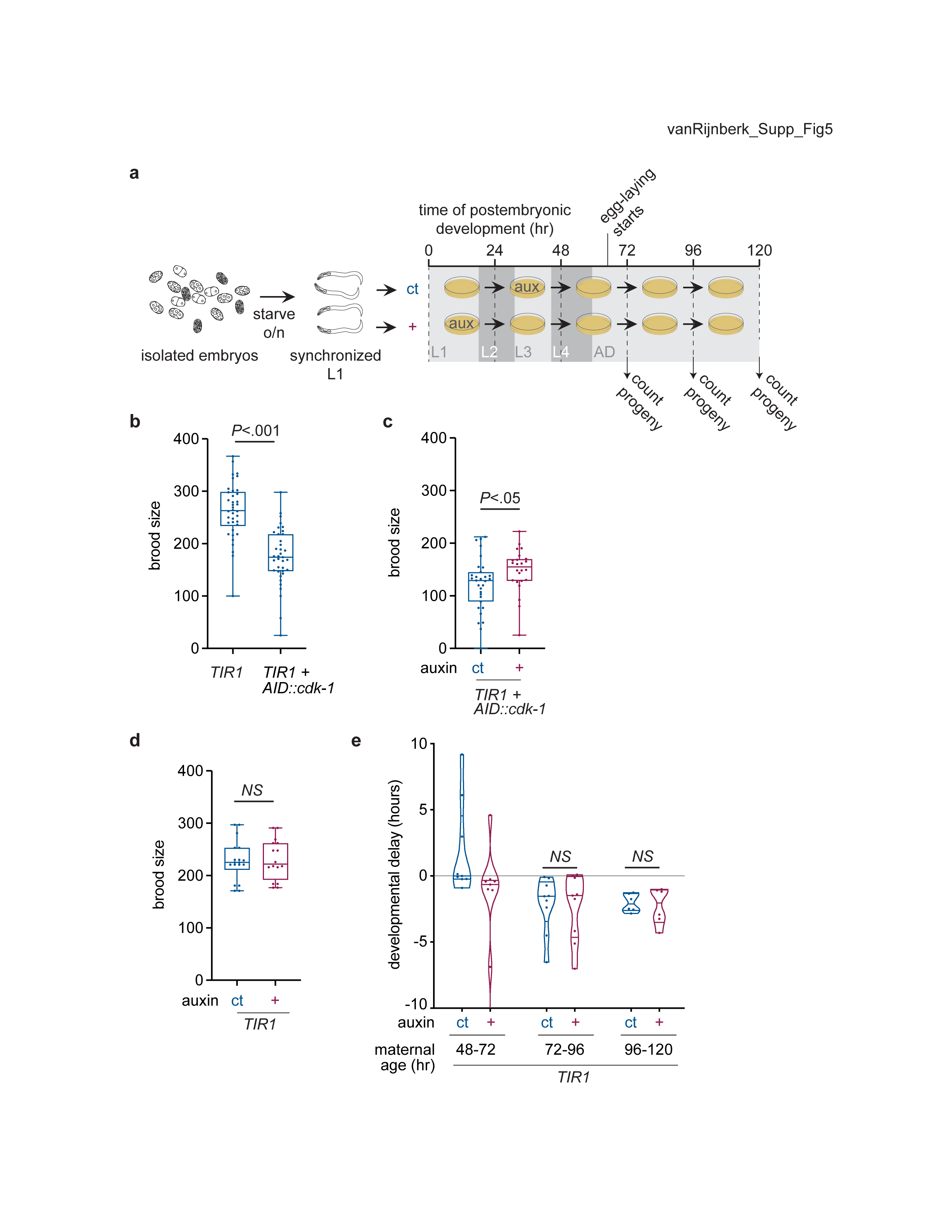

Supplement: S5 Fig — (A) Overview of experimental procedures for quantifications of brood size. Mixed stage embryos are isolated from adult hermaphrodites containing intestinal expressed TIR1 and either AID::cdk-1 or AID::knl-1, and starved overnight to yield a synchronized population of arrested L1 animals. The population of starved L1 animals is split into 2 conditions: an auxin (+) condition and a control (ct) condition. For the auxin condition, animals are grown on plates containing auxin for the first 24 hours of postembryonic development, when endomitosis normally occurs, and transferred to plates without auxin after this period. Animals in control conditions are grown on plates without auxin for the first 24 hours of development, and transferred to plates containing auxin for 24 to 48 hours of postembryonic development, when intestinal endomitosis has already occurred and neither KNL-1 or CDK-1 are required in the intestine. Worms are transferred to new plates at 24, 48, 72, 96, and 120 hours. Egg laying starts between 48 and 72 hours of postembryonic development, while virtually no eggs are laid after 120 hours of postembryonic development. After removal of animals from a plate, the remaining eggs are incubated for 16 to 18 hours to allow hatching, before progeny are counted for quantifications of brood size. (B) Brood sizes of Pges-1::TIR1 or Pges-1::TIR1; AID::cdk-1 animals grown without auxin. (C) Brood sizes of Pges-1::TIR1; AID::cdk-1 animals grown under control (ct) or auxin (+) conditions. (D) Brood sizes of animals expressing Pges-1::TIR1 and grown under control (ct) or auxin (+) conditions. Boxplots indicate the median and 25th to 75th percentile, error bars indicate min to max values, and individual values are shown as dots. P values were calculated by Mann–Whitney test. (E) Violin box plots depicting progeny growth rates of animals expressing Pges-1::TIR1 that were derived from mothers of different ages, and grown under control conditions (ct, n = 18 plates) or in the [file pbio.3001597.s005.tif]

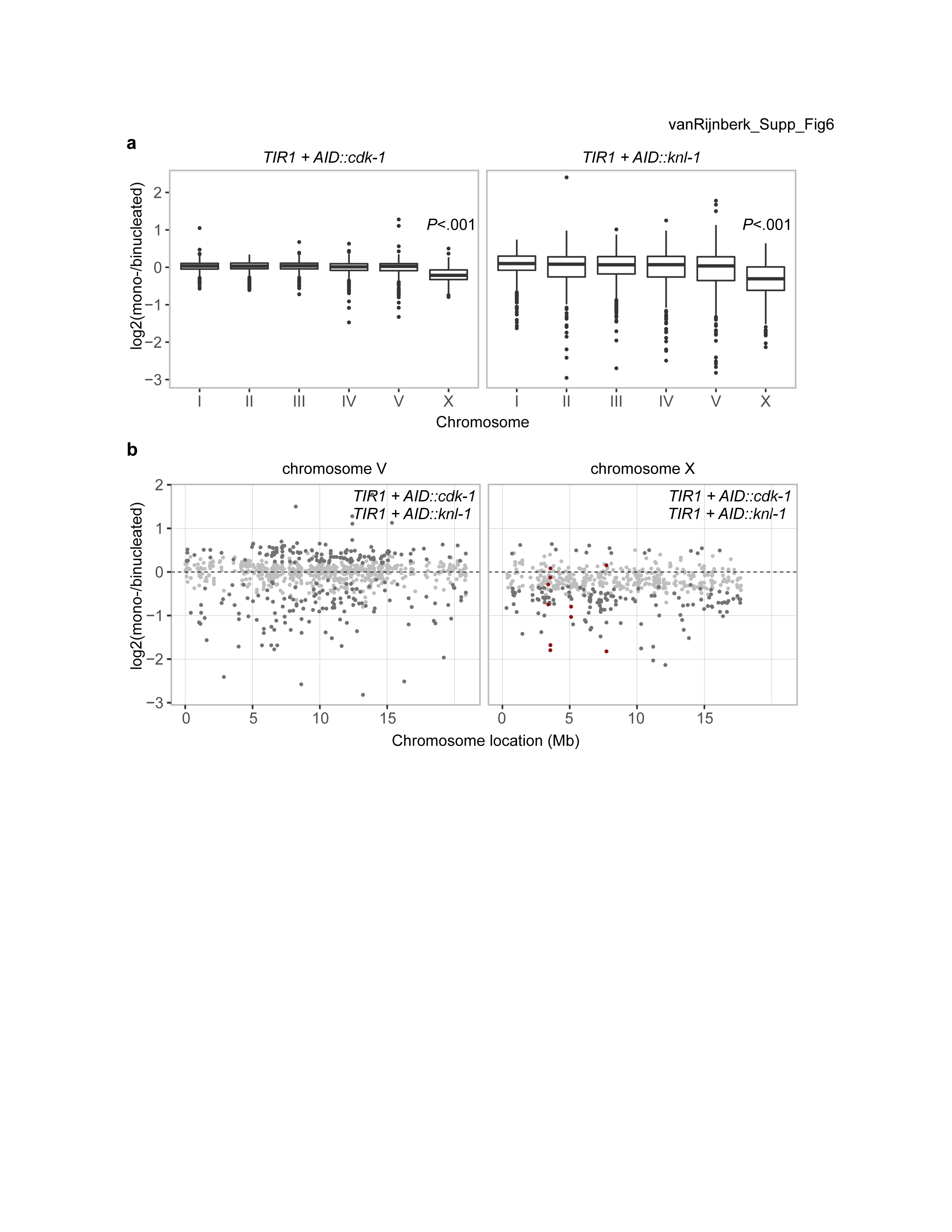

Supplement: S6 Fig — (A) Tukey boxplots showing the log2 fold change differential gene expression per chromosome in Pges-1::TIR1; AID::cdk-1 and Pges-1::TIR1; AID::knl-1 animals with a mononucleated versus binucleated intestine. P values of the comparison between X chromosomal and autosomal differential gene expression were calculated by Wilcoxon rank sum test. (B) Compiled log2 fold change of differential expression of genes located on chromosome V and X in Pges-1::TIR1; AID::cdk-1 and Pges-1::TIR1; AID::knl-1 animals with a mononucleated versus binucleated intestine. Data are compiled from single-worm RNA sequencing data of both Pges-1::TIR1; AID::cdk-1 and Pges-1::TIR1; AID::knl-1 strains. Genes with a top 25% percent absolute log2 fold change are indicated in dark gray, vitellogenins are indicated as red dots. Underlying data are available at the Gene Expression Omnibus, identifier GSE169330, and in S1 Data. AID, auxin-inducible degron. (TIF) [file pbio.3001597.s006.tif]

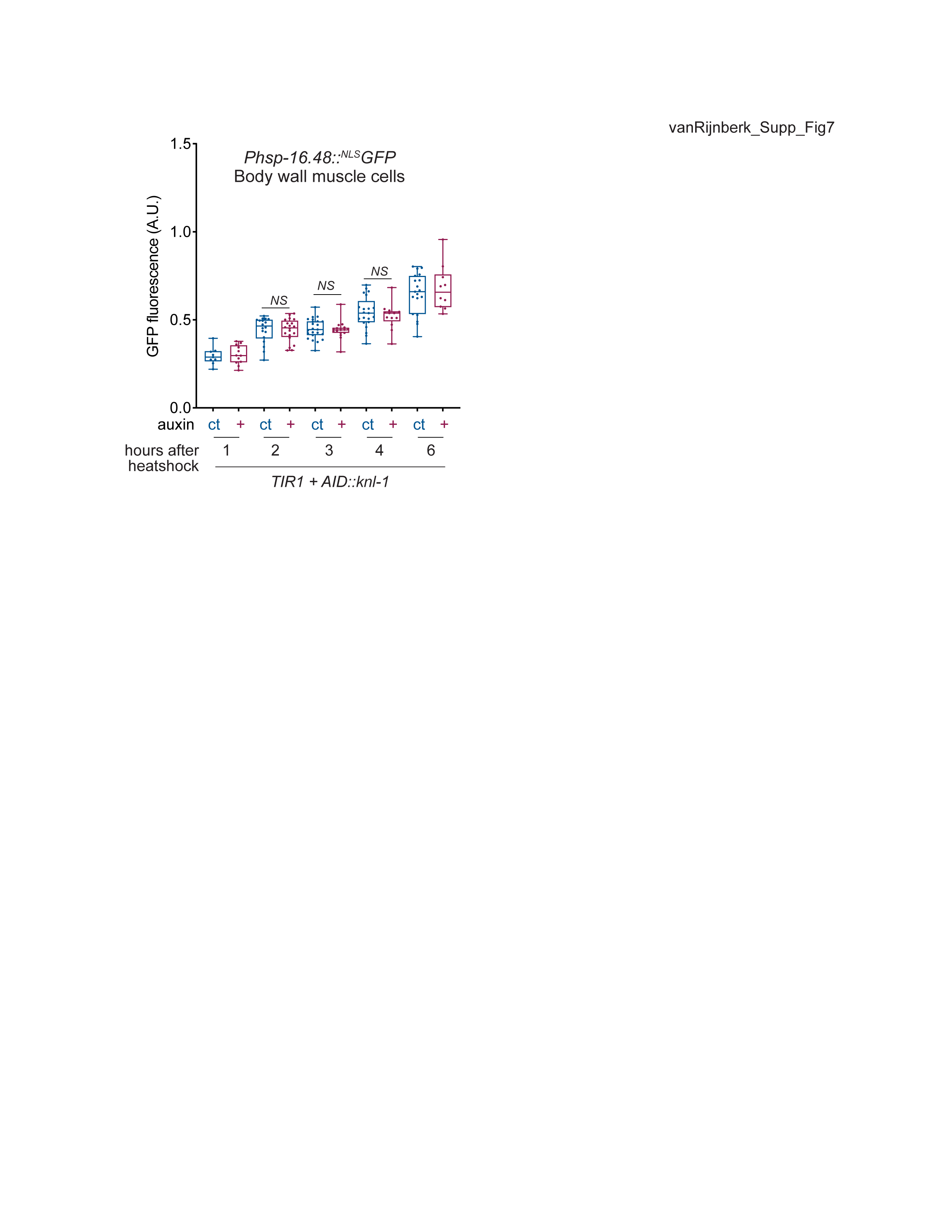

Supplement: S7 Fig — Boxplots with total nuclear fluorescence intensities of body wall muscle nuclei at different time points after heat shock in auxin-control (ct, n = 9 to 23) or auxin-treated (+, n = 10 to 20) Pges-1::TIR1; AID::knl-1; Phsp-16.48::NLSGFP animals, performed in 3 replicate experiments. Boxplots indicate the median and 25th to 75th percentile, error bars indicate min to max values, and individual values are shown as dots. P values were calculated by Mann–Whitney test. Underlying data can be found in S1 Data. AID, auxin-inducible degron. (TIF) [file pbio.3001597.s007.tif]

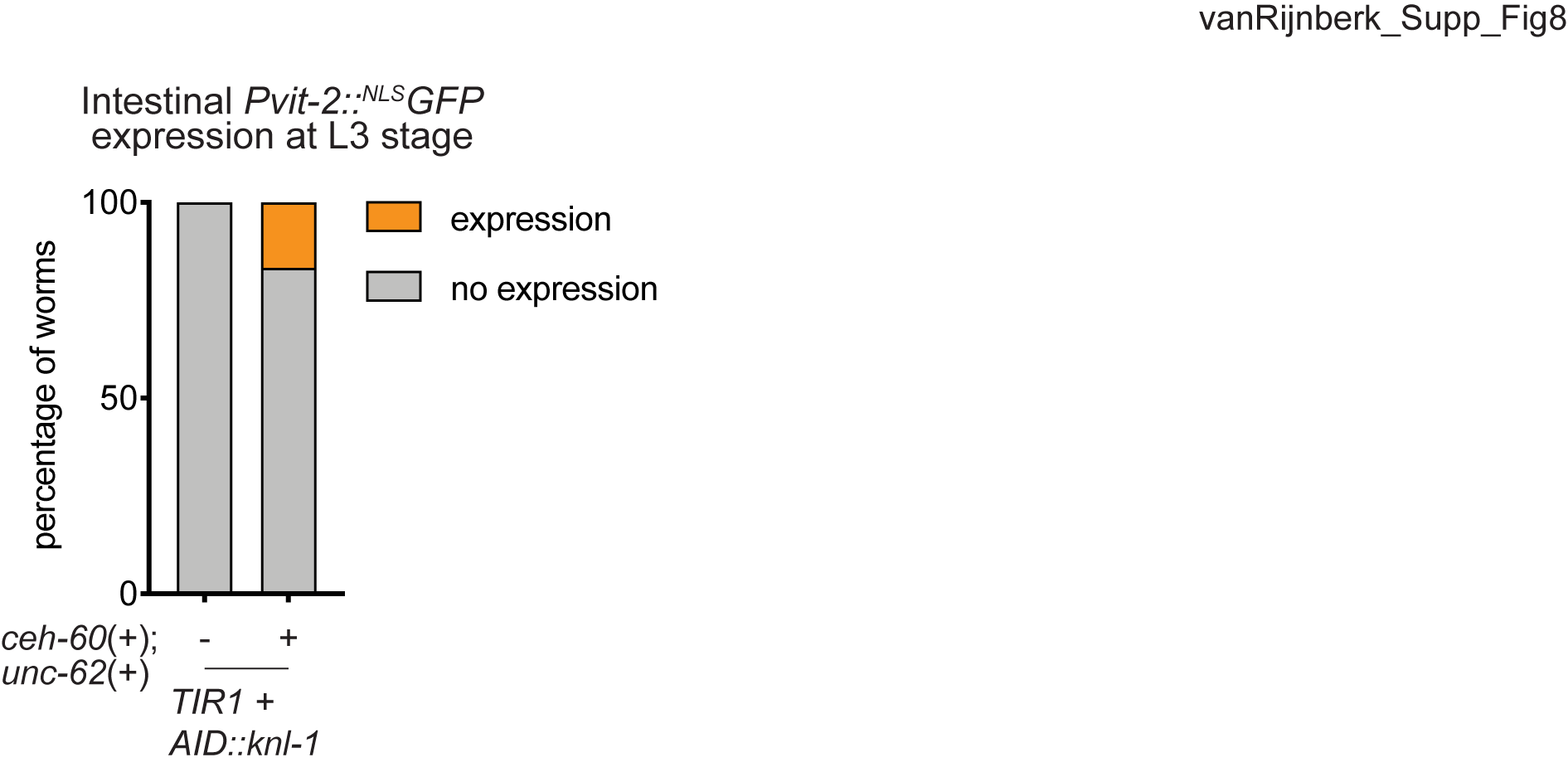

Supplement: S8 Fig — Bar graph depicting the percentage of animals with high levels of normalized total GFP fluorescence intensity per animal containing Pvit-2::GFP-NLS, for L3 animals with (+, n = 36) or without (−, n = 165) an intestinal overexpression of transcription factors ceh-60 and unc-62. A high level of total GFP fluorescence intensity is defined as more than twice the average control levels of GFP fluorescence intensity after background subtraction. Underlying data can be found in S1 Data. (TIF) [file pbio.3001597.s008.tif]
