# Supplementary material for: Endomitosis controls tissue-specific gene expression during development
Source: PLoS Biol. 2022 May 24;20(5):e3001597. doi: 10.1371/journal.pbio.3001597 (PMC9129049; doi:10.1371/journal.pbio.3001597)
Supplement: S1 Table — (PDF) [file pbio.3001597.s009.pdf]

Supplemental Table 1.

Strain name	Genotype	Source/ reference
BCN9071	*vit-2(crg9070[vit-2::gfp]) X.*	*Caenorhabditis* Genetics Center [25]
CA1209	*ieSi61 [Pges-1::TIR1::mRuby::unc-54 3'UTR + Cbr-unc-119(+)] II; unc-119(ed3) III*	*Caenorhabditis* Genetics Center [16]
GAL71	*ieSi61 [Pges-1::TIR1::mRuby::unc-54 3'UTR + Cbr-unc-119(+)] II; cdk-1(hu262 [AID::cdk-1]) III*	This study
GAL117	*knl-1(mat91[AID::KNL-1]) III; ieSi61 [Pges-1::TIR1::mRuby::unc-54 3'UTR + Cbr-unc-119(+)] II; unc-119(ed3) III*	This study
GAL126	*knl-1(mat91[AID::knl-1]) III; ieSi61 [Pges-1::TIR1::mRuby::unc-54 3'UTR + Cbr-unc-119(+)] II; unc-119(ed3) III; matIs53[Pges-1::sfGFP-PH::tbb-2 3'UTR; Pges-1::H2B-mCherry::unc-54 3'UTR; Plin-48::TdTom]] X*	This study
GAL137	*knl-1(mat91[AID::KNL-1]) III; ieSi61 [Pges-1::TIR1::mRuby::unc-54 3'UTR + Cbr-unc-119(+)] II; unc-119(ed3) III; vit-2(crg9070[vit-2::gfp]) X*	This study
GAL141	*matIs105 [Pges-1::Tir1-tagBFP]; matIs104 [Pact-5::npp-9-mCherry::tbb-2 3'UTR] II; knl-1(mat91[AID::KNL-1]) III*	This study
GAL160	*matIs114 [Pmyo-2::mCherry]; ieSi61 [Pges-1::TIR1::mRuby::unc-54 3'UTR + Cbr-unc-119(+)] II; knl-1(mat91[AID::KNL-1]) III*	This study
GAL162	*matIs116 [Pmyo-2::GFP]; ieSi61 [Pges-1::TIR1::mRuby::unc-54 3'UTR + Cbr-unc-119(+)] II; knl-1(mat91[AID::KNL-1]) III*	This study
GAL163	*knl-1(mat91[AID::KNL-1]) III; ieSi61 [Pges-1::TIR1::mRuby::unc-54 3'UTR + Cbr-unc-119(+)] II; matIs29 [Pges-1::CYB-1 DB::mCherry::unc-54 3' UTR; Pges-1::NLS-sfGFP::tbb-2 3' UTR; Pmyo-2::GFP]*	This study
GAL178	*knl-1(mat91[AID::KNL-1]) III; ieSi61 [Pges-1::TIR1::mRuby::unc-54 3'UTR + Cbr-unc-119(+)] II; unc-119(ed3) III; syIs44 [Phsp-16::lacI::GFP + lacO + dpy-20(+)] V*	This study
GAL182	*matIs115 [Pmyo-2::GFP]; ieSi61 [Pges-1::TIR1::mRuby::unc-54 3’UTR + Cbr-unc-119(+)] II; cdk-1(hu262 [AID::cdk-1]) III*	This study
GAL191	*matIs114 [Pmyo-2::mCherry]; ieSi61 [Pges-1::TIR1::mRuby::unc-54 3’UTR + Cbr-unc-119(+)] II; cdk-1(hu262 [AID::cdk-1]) III*	This study
GAL192	*matIs137[Pvit-2::NLS-sfGFP-AID_P2A_NLS-sfGFP-AID::tbb-2 3’UTR] II; ieSi61 [Pges-1::TIR1::mRuby::unc-54 3’UTR + Cbr-unc-119(+)] II; knl-1(mat91[AID::knl-1])III*	This study
GAL225	*matIs155[Phsp-16.48::NLS-sfGFP::tbb-2 3’ UTR]; knl-1(mat91[AID::knl-1])III; ieSi61 [Pges-1::TIR1::mRuby::unc-54 3'UTR + Cbr-unc-119(+)] II; unc-119(ed3) III*	This study

GAL226	*matIs137[Pvit-2::NLS-sfGFP-AID_P2A_NLS-sfGFP-AID::tbb-2 3'UTR] II; ieSi61 [Pges-1::TIR1::mRuby::unc-54 3'UTR + Cbr-unc-119(+)] II; knl-1(mat91)[AID::KNL-1] III; matEx151 [Pelt-2::ceh-60; Pges-1::BFP-P2A-unc-62; Pmyo-2::mCherry]*	This study
GAL246	*vit-5 (mat169)X; knl-1(mat91)[AID::KNL-1]III; ieSi61 [ges-1p::TIR1::mRuby::unc-54 3'UTR + Cbr-unc-119(+)] II; unc-119(ed3) III*	This study
GAL248	*vit-6 (mat171)IV; knl-1(mat91)[AID::KNL-1]III; ieSi61 [ges-1p::TIR1::mRuby::unc-54 3'UTR + Cbr-unc-119(+)] II; unc-119(ed3) III*	This study
GAL250	*knl-1(mat91)[AID::KNL-1]III; ieSi61 [ges-1p::TIR1::mRuby::unc-54 3'UTR + Cbr-unc-119(+)] II; unc-119(ed3) III; matIs172[Pelt-2::ceh-60::unc-54 3' UTR; Pges-1::BFP-P2A-unc-62(7a)::tbb-2 3' UTR; Plin-48::TdTomato]*	This study
GAL251	*vit-6 (mat171)IV; vit-5 (mat169)X; knl-1(mat91) [AID::KNL-1]III; ieSi61 [ges-1p::TIR1::mRuby::unc-54 3'UTR + Cbr-unc-119(+)] II; unc-119(ed3) III*	This study
TY5434	*syIs44 [Phsp-16::lacI::GFP + lacO + dpy-20(+)] V.*	*Caenorhabditis* Genetics Center [48]
