# Supplementary material for: Endomitosis controls tissue-specific gene expression during development
Source: PLoS Biol. 2022 May 24;20(5):e3001597. doi: 10.1371/journal.pbio.3001597 (PMC9129049; doi:10.1371/journal.pbio.3001597)
Supplement: S2 Table — (PDF) [file pbio.3001597.s010.pdf]

Supplemental Table 2.

Description	Sequence
cdk-1 N-terminal guide RNA target sequence	ataggatccataactaaaat
cdk-1 repair ssODN 1	ccttttcggcggcagtggaaatgattcaaaattacacgcttacgccttttctatcgttgtattacaattgttctgacaaaattcatttccAattttagttATGCCTAAAGATCCAGCCAAACCTCCGGCCAAGGCACAAGTTGTGGGATGGCCACCGGTGAGATCATACCGGAAGAACGTGATGG
cdk-1 repair ssODN 2	GCCACCGGTGAGATCATACCGGAAGAACGTGATGGTTTCCTGCCAAAAATCAAGCGGTGGCCCGGAGGCGGCGGCGTTCGTGAAGGATCCTATTCGCGAAGGAGAAGTGGCCCACGAGGGAGATTCGGTTTACACACTCAACGATTTCACGAAGCTCGAAAAAATCGGCGAAGGAACATACGGAG
knl-1 N-terminal guide RNA target sequence	cttacgaggctccatcgaca
knl-1 repair ssODN 1	tttattaccatttttaaaacatatttacagccatgCCTAAAGATCCAGCCAAACCTCCGGCCAAGGCACAAGTTGTGGGATGGCCACCGGTGAGATCATACCGGAAGAACGTGATGGTTTCCTGCC
knl-1 repair ssODN 2	GAGATCATACCGGAAGAACGTGATGGTTTCCTGCCAAAAATCAAGCGGTGGCCCGGAGGCGGCGGCGTTCGTGAAGggaggagccggagcatcgatggagcctcgtaagaagcggaactcgattct
vit-5 3' guide RNA #1 target sequence	tgcatctataaaaagggtaa
vit-5 3' guide RNA #2 target sequence	tttcagttgcatctataaaa
vit-5 5' guide RNA #1 target sequence	gcgtgtatttaaggcttcga
vit-5 5' guide RNA #2 target sequence	tttatttacgcgtgtattta
vit-6 3' guide RNA #1 target sequence	cgggtggctccgatgagggg
vit-6 3' guide RNA #2 target sequence	tggcgggtggctccgatgag
vit-6 5' guide RNA #1 target sequence	acaattgaaatgcaacaccg
vit-6 5' guide RNA #2 target sequence	tacaattgaaatgcaacacc
