# Supplementary material for: Endomitosis controls tissue-specific gene expression during development
Source: PLoS Biol. 2022 May 24;20(5):e3001597. doi: 10.1371/journal.pbio.3001597 (PMC9129049; doi:10.1371/journal.pbio.3001597)
Supplement: S3 Table — smFISH, single molecule fluorescence in situ hybridization. (PDF) [file pbio.3001597.s011.pdf]

Supplemental Table 3

smFISH probe sequences for *sfGFP* (28 probes)			
tggattagcttttcgacgtc	gcaagctttttagcattttc	caaccttgcgtttctttttc	agaattgggacaactccagt
cccattaacatcaccatcta	cctctccacggacagaaaat	taagggtgagttttccgttt	gtagttttccagtagtgcaa
agcattgaacaccataggtc	tcatgtgatccggataacgg	gggcatggcactcttgaaaa	agtgcgttcctgtacataac
tcccgtcatctttgaaagat	aaacttgacttcagcacgcg	gattaacaagggtatcacct	tcaataccctttaactcgat
agaatgtttccatcttcttt	agttgtactcgagtttgtgt	gccgtgatgtatacattgtg	gattccattcttttgtttgt
acggaaccatcttcaacgtt	gttgataatggtctgctagt	ccatcgccaattggagtatt	ggttgtctggtaaaaggaca
acagattgtgtcgacaggta	ttttcgttgggatctttcga	tcaagaaggaccatgtggtc	aatcccagcagcagttacaa
smFISH probe sequences for *vit-2* (46 probes)			
cacgagagaggcgatgatga	aaaggacgaggccatcgaac	catggagttcaaggaaggca	catgttgacaacagctctct
tttcttggagcaattgggtt	acttggtgatgatggtcttc	cggtgcacttatcgaagttg	cgtaggtgtagacagtttgt
tgacagtgtaaagggagcgg	tggttctcctcaagaacgag	gagtagatgatgctttcctt	tcttggatttgctcagtgat
ttggttccagcaatagcaag	agactcggcgaacttgatga	gaacgagtggacggatgttc	atctcgttgagttggttgac
ggtgtccttaagaagtctga	tcttgtagattgggaggagt	cattctccacaaagcaagca	ggagacaacttggacgagga
aacatgggttggtggacatg	cgatcatttgttcttgtggt	cgtgaagatcctttggcaag	gctgagagaatccgatttgg
aggctttcgagtttctcaag	attccagtttgaatacggcg	gagcacggatgttcattttc	ccttgtaacgaaggtagacc
tgctggtgaacttctcgata	ttgcggatagcttcgtagaa	tttggttctctggaacgaca	tcttcagcctcaatgtattc
acttcaagtccgaggaagtt	gttctttggattgttggagt	catgttacgctcgtatttct	tactctggcattccattgaa
atcattctcgttgttctctg	cagtaagttggtgcttgacg	gtcttcttggagagaacagc	ggagttgggcaacaatttct
tcagattggatcttctttcc	agtctctcgatttggtaagc	tcggtgttatcggaagtgta	ttctgtagttcttcttctcg
gaactccttgatttgtgtct	gaccttgacagactcaacga		
