# Supplementary material for: Endomitosis controls tissue-specific gene expression during development
Source: PLoS Biol. 2022 May 24;20(5):e3001597. doi: 10.1371/journal.pbio.3001597 (PMC9129049; doi:10.1371/journal.pbio.3001597)
Supplement: Attachment — Submitted filename: 116371_1_rebuttal_2248752_r45yf5.pdf [file pbio.3001597.s017.pdf]

Full Revision

Manuscript number: RC-2021-00761
Corresponding author(s): Galli, Matilde

1. General Statements [optional]

In this work we address the function of endomitosis, a common cell-cycle variation that leads to multinucleated polyploid cells. Although cells with multiple nuclei are common in many plant and animal tissues, it is largely unclear what the cellular advantages are of having more than one nucleus. Using the *C. elegans* intestine as a model system, we specifically manipulate intestinal endomitosis cell cycles that are required to generate binucleated cells, allowing us to compare mononucleate and binucleate cells that have the same cellular ploidy. Our study provides unique insights into the functional importance of binucleation for polyploid cell function: we find that animals with mononucleated instead of binucleated intestinal cells have defects in the upregulation of gene-expression during development, which leads to defects in the production of nutrients that are required for germline development, and ultimately gives rise to fitness defects in the progeny. Our findings demonstrate the importance of multinucleation for fine-tuning tissue-specific gene expression, revealing that the packaging of nuclear DNA into multiple nuclei can be beneficial to facilitate rapid transcriptional responses. Overall, the reviewers appreciate our work and agree that our study provides important insights into the function of multinucleation in polyploidy. The reviewers provided some valuable input to improve our manuscript and suggested some new experiments and textual changes, which we have performed and incorporated in the revised version.

2. Point-by-point description of the revisions

Below, we have pasted the review reports for our manuscript (in black), with our responses and and revisions shown in blue:

Reviewer #1 (Evidence, reproducibility and clarity (Required)):

One of the mysteries of polyploidy is how a cell's ploidy state (mono, bi, or multinucleated) affects its function. This study uniquely develops an intestinal model in C. elegans to address this long-standing question. The worm intestinal cells are usually binucleated with 2, 32C nuclei, but authors constructed strains with mononucleated 64C cells by inhibiting progression through the cell cycle. As result, the worm and its intestine developed and grew to its normal size, but with mono- instead of binucleated polyploid intestinal cells. The authors then characterized how loss of binucleation affected C. elegans fitness and gene expression identifying a reduction in vitellogenin genes leading to reduced lipid loading in the oocyte. Further investigating the transcriptional regulation using heat shock response, the authors discover that binucleation is necessary for rapid upregulation of heat shock transcripts. This study reveals how the binucleated ploidy state may be advantageous for gene expression regulation.

Review COMMONS

Overall, this is a timely study given the recent interest in how ploidy state affects many mammalian cell types including hepatocytes and cardiomyocytes. And the conclusions are well supported by the data, but it seemed odd that authors focused on non-cell autonomous effects of intestinal ploidy. In particular, gene expression was assayed in single worms instead of single mono- versus binucleated polyploid intestinal cells.

The reason why we used whole worms instead of single intestinal cells for the RNA sequencing experiments is because we are currently unable to dissociate single intestinal cells from adult *C. elegans* as these cells are connected by strong junctions that are difficult to disrupt without rupturing the cell. However, because intestinally expressed genes have already been defined in previous studies, and because the intestine is one of the most transcriptionally active and largest tissues in the worm, making up roughly one-third of the animal's volume, we are able to deduce how mononucleation/binucleation affects intestine-specific gene expression by whole worm sequencing. We have added extra information about the RNA sequencing procedure and how we determined intestinally expressed genes in the Methods section, see page 23/24.

Major comments:

1) The authors should verify the reduction of vitellogenin gene expression in whole animal is due to a reduction in vitellogenin transcripts in the mononucleated intestinal cells by smFISH or another method. This was only shown for heat shock response, yet it is assumed, but not demonstrated to be the case in single worm studies.

We have performed *vit-2* smFISH experiments in mononucleated and binucleated intestinal cells and find that mononucleated intestinal cells have on average lower amounts of *vit-2* mRNAs per cell. We also quantified the total number of nascent vit-2 transcripts in intestinal nuclei and find that mononucleated cells have significantly lower number of nascent transcripts per cell, which is consistent with our RNA sequencing data. We have included this new data in the revised version, see Figure 4B-D and page 9 and 10 (lines 207-213) of the revised version, as well as the updated Methods section, where we describe how we performed the analyses using FISH-quant (Page 26-27, line 620-627).

2) Figure 7 shows transient activation of vitellogenin expression rescues vit-2 downregulation in animals with mononucleated intestines. If this is case, then authors should test whether transient activation of vitellogenin expression is sufficient to rescue reproductive fitness and oocyte lipid loading as assay in Figure 3 and 5.

We have generated a new strain that overexpresses the CEH-60 and UNC-62 transcription factors. In this strain we find that lipid loading into embryos is increased (Figure 7C), which is expected because CEH-60 and UNC-62 drive the expression of vitellogenins that in turn are used for lipid loading into developing oocytes. Importantly, in this strain we no longer see a defect in the progeny of animals from mononucleated mothers: the amount of lipids in embryos and the

brood sizes are now very similar between the progeny of animals that have either a mononucleated or binucleated intestine (Figure 7C,D). With these new results, we show that transient activation of vitellogenin expression is sufficient to rescue reproductive fitness and oocyte lipid loading. We have included this data in the revised version, see page 15 and 16 (line 344-364).

Minor comments:

3) In Figure 2, representative images should be shown with each graph on intestine, cell, and brush broad sizes that was measured.

We have added representative images of the intestinal cells and lumen/brush border of animals in Figure 2C and 2F.

4) The discussion should put this work in context with what is known and unknown in the field. Several studies have examined ploidy state and its effect on gene expression as well as other cell/tissue properties. These references include, but are not limited to, cardiomyocytes (Windmueller et al. Cell Reports 2020), hepatocytes (Ow et al. Elife 2020), and multinucleated skeletal muscle (Petrany et al. and Dos Santos et al. Nat. Commun, 2020).

We have added a paragraph in the discussion to describe what is known about how multinucleation influences transcription and other cellular properties, and how our work relates to this:

Page 16, lines 373-388:
"Multinucleated cells are present in many different animal tissues, however it is largely unknown why certain cells become multinucleate and how this influences gene expression. Recent studies have begun to investigate the transcriptional activities of multinucleated cells such as mouse cardiomyocytes, hepatocytes and skeletal muscle cells (16, 41-44). These analyses have revealed gene expression changes that occur in multinucleated cells, however it is unclear from these studies whether these transcriptional changes are a direct consequence of multinucleation, or arise in response to differentiation cues and are thus only indirectly associated with multinucleation. For example, binucleate cardiomyocytes were shown to have reduced expression of cell-cycle genes and an increased expression of genes involved in cardiomyocyte maturation, suggesting that multinucleated cells proliferate less and are more differentiated (16). Nonetheless, it is unclear whether multinucleation per se influences the expression of cardiomyocyte maturation genes. This could be tested by comparing gene expression profiles between multinucleated 4N cells and mononucleated 4N cells, which is technically challenging because the majority of polyploid murine cardiomyocytes are multinucleated (16)."

5) The order of data/figures seems out of place and suggest discussing gene expression results first then reproductive fitness.

We agree with the reviewer that it is better for the flow of the story to switch the order of the results and show the gene expression changes first and then show that the animals have reduced reproductive fitness, which is consistent with a decrease in vitellogenin expression. We have applied this change in the revised version.

6) Terminology correction: endoreplication encompasses both endocycle and endomitosis (Edgar et al. Nat Rev Mol Cell Biol., 2014). The generation of mononucleated intestinal cells described in this study then occurs by the endocycle and endomitosis (specifically failed karyokinesis).

There is quite some confusion regarding the naming of different types of polyploidization cycles – The term "endocycles" is especially confusing because it has been used to describe both endoreplication and endomitosis. Overall, "endoreplication" is usually used to describe cell cycles in which cells alternate between S phase and Gap phase, and "endomitosis" to describe cell cycles in which cells undergo all canonical phases of the cell cycle (G1/S/G2/M) but do not complete M phase. We have therefore decided to adhere to these terms, and not mention the word "endocycles".
In the review by Edgar et al, to which the reviewer refers to, the terms "endoreplication" and "endocycles" are used interchangeably, and as this review does not focus on endomitosis regulation, the distinction between endoreplication and endomitosis is not made. However, in another review by the same authors (Zielke et al, Cold Spring Harb Perspect Biol. 2013) this distinction is clearer and is in line with the terminology that we use in our manuscript.

Reviewer #1 (Significance (Required)):

This study identifies how binucleation influences gene expression by rapidly inducing transcription. The effect of ploidy on gene expression has been examined in other cell types and model systems with mixed results, some finding significant changes and others not. The advantage of this study is that cell ploidy is equal between the mono and binucleated cells generated and the effects of each ploidy state can be assayed in vivo. Overall, the authors' discovery that binucleated intestinal cells are necessary for animal vitellogenin expression and reproductive fitness will be of wide-interest to researchers in both the polyploid and reproductive fields.

Reviewer #2 (Evidence, reproducibility and clarity (Required)):

In this paper by van Rijnberk et al, the authors set out to understand the functional significance of endomitosis, a phenomenon where mitotic chromosome partitioning is not followed by cytokinesis, which results in cells with two nuclei. Using C. elegans intestine divisions as a model for endomitosis, the authors developed means to disrupt endomitosis in a tissue-specific manner. They next analyze the functional consequences of loss of endomitosis on post-

embryonic development and gene expression. The approach developed in this paper is robust and well controlled and the findings will likely influence studies aiming to understand endomitosis in other systems. Unfortunately, most of the observed phenotypes are quite weak or not significantly different from the control. Therefore, this paper would greatly benefit from strengthening analysis of a couple of the most robust phenotypes.

Major Comments:

1) One of the conclusions of the study, as stated in the abstract, is that "reduced expression of vitellogenins in mononucleated intestinal cells leads to progeny with developmental delays and reduced fitness". However, the authors have not conclusively demonstrated that their phenotypes are a direct consequence of reduced vitellogenin expression in the intestine. To conclude this, the authors would need to either test if reducing the expression of vitellogenins in intestine cells can phenocopy loss of endomitosis, which might be technically challenging since there are 6 redundant vitellogenin genes, or test if overexpression of one or more vitellogenins can rescue the observed phenotypes.

We have generated single knock-outs of two of the six vitellogenin genes (*vit-5* and *vit-6*) to test whether they have reduced brood sizes, which is one of the phenotypes that we observed in the progeny of animals with mononucleated intestinal cells. Indeed, we find reduced brood sizes in *vit-5* and *vit-6* mutants, and even further reduced brood sizes in *vit-5;vit-6* double mutants, demonstrating that reduced vitellogenin levels leads to the same phenotype as blocking endomitosis. These new results are described on page 14 and 15, lines 328-335 and are shown in Figure 7A.

We have not attempted to overexpress individual vitellogenins, however we have overexpressed the transcription factors that drive their expression (CEH-60 and UNC-62) and find that this can rescue the defects arising from mononucleated intestinal cells (see our response to the next point for more information).

2) One of the most compelling results is that, upon loss of endomitotic cycles in the intestine, there is a significant decrease of lipid accumulation in the embryos (Fig. 5c), which correlates with reduced vitellogenin expression. The authors should pursue this result further to test if this defect is indeed due to loss of vitellogenin expression in the intestine and to investigate further the consequences of loss of lipid accumulation in embryonic/post-embryonic development. The authors should also test if the reduced lipid accumulation in embryos correlates with maternal age.

This point is very similar to point 2 of reviewer #1, and point 13 of reviewer #3, namely to provide more evidence that inhibition of endomitosis leads to reduced fitness due to a decrease in vitellogenin expression and lipid loading into embryos. To address this, we have generated a strain that overexpresses the transcription factors that drive vitellogenin expression (CEH-60/UNC-62) and find that it can rescue the lipid loading and brood size defects in the progeny of

animals with mononucleated intestinal cells. These new results are shown in Figure 7C,D and are described on page 15 and 16, lines 344-364. Together, these new results provide additional evidence that blocking endomitosis leads to reduced vitellogenin expression, reduced lipid loading into embryos, and reduced brood sizes in the progeny.

3) As stated above, many of the phenotypes reported in the paper are very mild (e.g. Fig. 3g, 3h, 5a, 5b, 6a, 7) and in the range where their significance is debatable. Therefore, the authors should be careful to qualify conclusions when describing the results in the text.

We agree that the phenotypes that we observe in animals with mononucleated intestinal cells (and their progeny) are very mild, and we understand that questions can arise regarding their significance. To make it clearer which differences are statistically significant and which ones are not, we annotated the non-significant differences as “NS” (not significant) in the figures.

We would like to stress that, although the differences in developmental timing and brood size are small, the impact they have on reproductive fitness is substantial. We have made a textual adjustment to make it clearer that the brood size differences are not very big:

Page 12, lines 269-271:
“Moreover, animals derived from mothers with mononucleated intestines showed a mild decrease in brood sizes compared to controls”

Furthermore, we removed the embryonic viability data, as we agree that the differences here were very small.

In the experiments with the *Pvit-2::GFP* and VIT-2::GFP strains, our focus is on the decrease in vitellogenin promoter activity and vitellogenin expression at 72 hours of development, which is substantial, very significant and highly reproducible between replicate experiments. In the revised version, we have made textual adjustments to clarify this:

Page 9, lines 204-207:
“This difference in vit-2 expression levels is no longer present in older adults, when vit-2 expression is peaking and becomes similar between animals with mononucleated and binucleated intestinal cells (Figure 4A, 96 and 120 hour timepoints).”

Finally, we have performed additional brood size calculations, as well as lipid loading experiments (similar to those shown previously in Figures 3G, 5B and 7) in the presence and absence of the UNC-62/CEH-60 overexpression as well as in *vit-5*; *vit-6* mutants with either mononucleated or binucleated intestines. The results of these new experiments (Figure 7A,C,D of revised version) are consistent with our previous findings, further strengthening our conclusion that the effects on reproductive fitness are mild, but reproducible and significant.

4) Some experiments need clarification in terms of their execution. For instance, in the reproductive fitness assay in Fig. 3a-c, at which generation or plate dilution number was the ratio measured to generate the graphs? Also, in figure 3f, it was not clear from the methods how the developmental delay was estimated in these experiments and what control was used to determine if a delay was observed or not.

We have clarified the experimental procedures in the figure legend of Figure 5 (former Fig.3) to clarify at which plate dilutions the reproductive fitness measurements were made, and how the developmental delay was calculated.

Reviewer #2 (Significance (Required)):

Endomitosis and endoreduplication are prevalent across species. This manuscript presents a clean approach to inhibit endomitosis and address the consequences. The effects reported are quite mild but a bit more focus on a couple of key observations should lead to an interesting and impactful manuscript.

Reviewer #3 (Evidence, reproducibility and clarity (Required)):

In the manuscript entitled "Endomitosis controls tissue-specific gene expression during development", van Rijnberk and colleagues assess the functional significance of multinucleation in normally polyploid cells. Specifically, the authors assessed changes in gene expression when normally binucleated polyploid intestine (32n) cells in C. elegans are forced to develop as mononucleated (32n) cells without affecting genome ploidy. For this study, the authors developed a method to prevent binucleation without affecting polyploidization of intestinal cells by transiently inhibiting specific regulators of mitosis using the Auxin-inducible degradation system. They find that binucleation is necessary for the expression of intestinal genes. Particularly, mononucleation resulted in the downregulation of genes that normally increase in expression rapidly during the larva to adult transition. These DEGs include the Vitellogenin yolk proteins that are involved in the transport of nutrients to the germline. The authors concluded that binucleation of intestinal cells is required for the normal expression of intestinal genes and for animal fitness.

General comments:

The paper is generally well written, but it is still difficult to read as important experimental design details are either absent or buried. Authors also overstate the role of nuclear organization on the changes in gene expression observed when normally binucleated cells are forced to be mononucleated. Usually, nuclear organization refers to the location of specific chromatin regions of the chromatin within the nucleus. Although the nuclear volume changes in size the cell size

doesn't in the mononucleated cells, the authors do not specifically show that the chromatin within the nucleus is organized differently than in binucleated cells. In addition, other than nuclear organization effects that can change gene expression are also expected (e.g. nuclear transport capacity) or observed by the authors (e.g. the nuclear volume to cytosol volume ratio) in mononucleated intestinal cells. See Mary K Baylies publication in Drosophila muscle cells. doi: 10.1242/dmm.011361. Epub 2013 Apr 4.
Also, in some sentences, it is unclear what the authors are referring to or lack in experimental/data details (see specific comments below).

We agree with the reviewer that it is confusing that we talk about nuclear organization differences between animals with mononucleated or binucleated cells, as the term "nuclear organization" is mostly used in the context of chromatin localization within the nucleus. In the revised version, we have replaced *"nuclear organization"* with *"genome organization"* (page 16, line 372) and also added an additional paragraph in the conclusion about what is known on how multinucleation affects gene expression (page 16, lines 373- 388, see also our response to reviewer #1, point 4).

Specific comments:

1) Although the authors say they expressed TIR under an intestine-specific promoter, they do not clearly state the promoter used. Please include the specific promoter's name used to express the degrons in the main text, where it can be accessed easily by the reader. If they used ges-1 promoter, as it appears from the method section, this promoter is also expressed in the pharynx and head mesoderm in addition to the intestine.

In our experiments, the TIR1 is indeed expressed using the *Pges-1* promoter: we have added this information in the revised version. The *Pges-1* promoter is commonly used for intestinal-specific expression and Zhang and colleagues (2015) have demonstrated that, when used to drive TIR1 expression, it confers intestinal-specific degradation with the auxin-inducible degradation system. Consistent with this, we see that expression of *ges-1* in (unpublished) spatial transcriptomics data generated by our lab is limited to the area of the intestine and absent in the pharynx and head mesoderm. Finally, even if there would be some leaky expression of *ges-1* in the pharynx and/or head mesoderm, this would not interfere with our analyses as it is known that the pharynx and head mesoderm are fully formed during embryogenesis and do not undergo any divisions in larvae. As we only grow animals in the presence of auxin during larval stages, it is highly unlikely that inhibition of CDK-1 or KNL-1 would have an effect on these tissues.

To make it clear that we have used the *Pges-1* promoter in our experiments, we have included this information in the text on page 5 (line 89-90):

"By using a strain that expresses TIR1 under control of the intestine-specific Pges-1 promoter and exposing animals to auxin only during the time that endomitosis occurs, we can specifically inhibit intestinal binucleation without affecting canonical cell cycles of intestinal or other cells"

2) The authors state: "To control for non-specific effects of auxin on development, we included an auxin control in each experiment, in which animals were treated with auxin for the same duration, but during development in which CDK-1 or KNL-1 are not required". This should read "... are not required in the intestine" if the authors are a) positive that TIR is not being expressed at all anywhere else and b) there are no other tissues during the developmental stage of auxin exposure that are undergoing other cell divisions requiring CDK-1 or KNL-1.

We have added *"in the intestine"* to this sentence (see page 5, line 100 of revised version) to make it clearer that CDK-1 and KNL-1 are only degraded in the intestine at a specific timepoint.

3) Controls: It is not clear why the authors did not include the minus (-) control in some of the assays (e.g. animal length and width in figures 2a, b, i, j, k).

When we first started the experiments, we only used the minus (-) condition (so no auxin at all) as a control condition. We later decided that the auxin-control (ct) condition was a better control because it would also control for possible (side) effects of auxin on development. Therefore, many experiments have both "-" and "ct" control conditions, however there is no particular reason that the "-" condition was not included in some experiments. To avoid confusion and to make our findings clearer, we have decided to remove the "-" conditions from all the figures and text, as they do not add any relevant information.

4) Authors should justify why they did not include control nematodes that have the AID system but lacking the degrons on KNL-1 and CDK-1 treated with auxin in their experiments. In multiple experiments this control would have been more relevant than the nt control, see 5) below as an example of this. The authors do mention a difference in brood sizes and embryonic lethality in the AID::CDK-1 compared to the wild-type animals. Neither this data nor quantification of this data is shown in text or figures. Because the strain with the AID tag on CDK-1 is a hypomorph, the AID strain without a degron might be a very important control in all experiments.

In the revised version, we have included the data that shows the reduced brood size in the strain with the CDK-1 degron, see Supplemental Figure 5B.

We agree with the reviewer that the analysis of a strain that contains the AID system but lacks the KNL-1 and CDK-1 degrons is important for the interpretation of our results. We would like to point out that we have characterized this strain, see Supplemental Figure 5D,E, and lines 267-269 on page 12: Here we have quantified brood size, embryonic lethality and developmental timing in a strain containing the AID system but lacking the KNL-1 and CDK-1 degrons, and find that there is no difference whether these worms are grown in the control (ct) or auxin (+) condition.

Therefore, we are convinced that the defects that we observe when we perform the same experiments in the presence of the KNL-1 or CDK-1 degrons are due to blocking endomitosis.

5) It is not clear which control is being used in the fitness assay Figure 3a or main text, one might have to go through strain tables/supplemental/methods sections in detail to find out. This is important because the nt control would not be appropriate for this assay. Either +/- auxin of the test strains or + auxin of the strain with a strain containing the AID system but without the degron for CDK-1 or KNL-1 would be better controls than nt (see 4).

We agree with reviewer that using a no treatment control would be inappropriate for this assay: indeed, we used the auxin-control (ct) condition to generate binucleated animals for the relative fitness assay. In the revised version, we have removed all experiment with the "-" condition, so it should now be clearer which control was used in this experiment. Also, we adjusted the text to make it clearer:

Page 11, line 241-244:
"By mixing equal amounts of animals with mononucleated (auxin-treated, +) or binucleated intestines (control condition, ct) on plates and counting the proportion of GFP and mCherry positive progeny after several generations,…"

The reason that we did not perform the relative fitness assay with the strain lacking the CDK-1 or KNL-1 degrons is because we do not see any differences between animals grown on auxin (+) versus animals grown on auxin-control (ct) conditions in this strain (see supplemental Fig. 3). Therefore, we do not expect any differences to arise in relative fitness due to these conditions in the absence of the KNL-1 or CDK-1 degron. Because the relative fitness assays require a substantial amount of time (two to three months if everything goes well), we would prefer not to carry out these experiments.

6) The n for the animal with and length seems a bit small given the variability observed (please specify how the n was chosen to be about 15 animals for this assay).

We agree with the reviewer that the n value was rather small for these experiments – we have performed additional experiments and added more data points in these figures (Figure 2A, B).

7) The authors do not provide information about the efficiency of their method. Do all experimental auxin-treated animals result in progeny with mononucleated polyploid cells?

We regret that we forgot to mention the efficiency of our method in the manuscript. In our experiments, we see that mononucleation of intestinal cells occurs in all auxin-treated animals. We have added a quantification of the cell/nuclei number in control versus auxin conditions in Figure 1E,G and described it in the main text:

Page 5, line 105-107:

"Of the 12-14 intestinal cells that are known to undergo endomitosis and become binucleated in wildtype intestines, we found on average 13/14 cells to be binucleated in auxin-control animals compared to 0/14 binucleated cells in auxin-treated animals (Figure 1F,G)."

8) Figures:
Figure 1a: Mitosis label is hard to see in some of the cell division/endomitosis and endoreduplication cycles because they are in the same color as the cycle arrows.
Figure 1b and S1a, it is not clear why there are arrows between each plate in the ct control only.
Figure 3a: It is not clear what "dilute" refers to in the schematics
Figure 9S does not exist and is really Figure 8S.
Figure 1S: Where they state "... subsequent endoreplicative cycles in the first and second larval stage for 60 to 200 worms per condition per timepoint, in three replicate experiments. X axis starts at 16 hours". Please specify the 16hrs post-hatching, post egg-laying or post-fertilization? Either way at any temperature this does not match to L1 and L2 hours of development. Also, are the differences in each time point statistically significant? Why are there no error bars at some time points?

In response to the reviewer's comments and questions regarding specific figures and figure legends, we have made several adjustments:

- We have adjusted Figure 1 to make the cell cycles and developmental progression clearer
- In Figure 1C (previously Figure 1B) and Supplemental Figure 5A, we have added additional arrows to clarify that animals of each condition were transferred to new plates.
- In the figure legend for Figure 5A (former Figures 3A), we have added a short explanation of the experimental procedures performed to dilute the population of animals.
- We have removed the erroneous reference to the non-existent Supplemental Fig. 9, and correctly referenced Supplemental Fig. 8.
- In the experiment shown in Supplemental Figure 1E, we synchronized L1 animals by overnight starvation, let them grow on auxin or control plates and imaged them at various timepoints between 16 and 30 hours of development at 15 ºC. Within this timeframe, we are able to capture endomitosis G2/M phase (around 20 hrs of development at 15 ºC), and the endoreplication S phase that takes place at the end of L1 (around 24 hrs of development at 15 ºC). We realize that we had made a mistake in the main text (we said that we looked at multiple endoreplicative cycles), we apologize and thank the reviewer for pointing this out. We have corrected this sentence in the revised version (see page 6, line 131-132). Additionally, we have changed the X-axis in the graph and made some textual changes in the figure legend to make the timing clearer. Regarding the statistical significance and error bars: we do not have replicate experiments for each timepoint, hence there are no error bars for all points. We have added this information in the figure legend. We also added P values in the graph: although some timepoints are statistically different between auxin and control animals, we do not see large differences in overall timings.

9) Auxin treatment details missing or hard to understand, please clarify so that reader does not have to guess based on bits of information throughout the manuscript. Specifically, Figure 1b and Figure 3S schematic does not match the methods (which are incomplete regarding the

assay). From worm culture in methods: "synchronized L1 larvae were divided over three possible conditions: no auxin (-), auxin control (ct) and auxin (+). Auxin worms were grown on auxin for 24 hours, after which they are transferred to control plates. Auxin control worms were grown on control plates for 24 hours and transferred to auxin plates from 24 to 48 hours of development." What I understand from this and the figure captions for both Figures is that AID-degron (for CDK-1 or KLN-1) worms grown at 20 degrees Celsius were exposed to auxin since L1 from 24 hrs to 48 hrs. Either the authors meant that these experimental worms were exposed to auxin after arrested being arrested overnight for 24hrs and nt worms were exposed to auxin starting at 24 hrs post L1 arrest and ending 24 hrs later. In both nt and the + worms were exposed for 24 hrs to auxin, but the nt worms were exposed from the end to the L1 stage to mid the L2 stage and the + worms were exposed from the L1 arrest to a few hours before the L2 molt.

We realize that the schematic of the experimental conditions depicted in Figure 1 and Supplemental Figure 5 (former Supplemental Figure 3) was not completely clear. In the revised version, we have altered the axis in the figures to indicate that we indeed are referring to the time of postembryonic development and we have provided a better explanation of the experimental procedures in the figure legend of Figure 1B:

Page 37, line 830-840:
“Schematic overview of experimental procedure. Mixed stage embryos are isolated from adult hermaphrodites containing intestinally expressed TIR1 (Pges-1::Tir1) and either AID::cdk-1 or AID::knl-1, and starved overnight to yield a synchronized population of arrested L1 animals. The population of starved L1 animals is split into two conditions: an auxin (+) condition and a control (ct) condition. For the auxin condition, animals are grown on plates containing auxin for the first 24 hours of post-embryonic development, when endomitosis normally occurs, and transferred to plates without auxin after this period. Animals in control conditions are grown on plates without auxin for the first 24 hours of development, and transferred to plates containing auxin for 24 to 48 hours of postembryonic development, when intestinal endomitosis has already occurred and neither KNL-1 or CDK-1 are required in the intestine”

Also, as we mention in response to point 3 of the reviewer, we have removed the no auxin (-) condition from our analyses, which has also simplified the schematics.

10) The first sentence at the top of the result title binucleation is important for adult intestinal transcription, proposes the following hypothesis "We hypothesized that the observed fitness phenotype may be caused by transcriptional changes of the intestine caused by an alteration of the nuclear size or surface-to-volume ratio." However, the differential gene expression will not actually test this directly. So it is slightly misleading.

We acknowledge that this sentence was slightly misleading so we have removed this sentence. In addition, we have switched the order of the results section in response to reviewer #1, point 5 – we now describe the transcriptional results first, then the reproductive fitness results.

11) In the same section as in 10), the authors say "AID::cdk-1 animals revealed fewer differentially expressed genes between animals with mononucleated or binucleated intestines, which is likely due to a lower complexity of these samples." It is not clear why the AID::cdk-1 samples are of lower complexity.

Unfortunately, we do not know why we found lower complexity of RNA samples from animals with an *AID::cdk-1* background. One explanation could be that RNA degradation took place in these samples, more so than in samples with *AID::knl-1* animals, because the *AID::cdk-1* samples were kept in the freezer for a slightly longer period of time before downstream processing. However, storing samples in the freezer has not been an issue previously, so we're not sure why we found lower complexity in these samples. Nonetheless, we still think it is valuable to include the *AID::cdk-1* data in the manuscript, and focus on the overlap between differentially expressed genes in animals with the *AID::knl-1* and animals with the *AID:cdk-1* background. Because of the lower complexity in the *AID::cdk-1* sample, this approach may be a little stringent and we could miss genes for which expression differences are subtle, but it still allows us to extract the main and more consistent expression changes associated with mononucleation of the intestine.

To make it clearer that we do not know why we find lower complexity in the AID::CDK-1 samples, we included this sentence:

Page 8, line 173-177:
"Although it is unclear why the AID::cdk-1 samples had lower complexities, we focussed on the overlap in differential gene expression between the AID::knl-1 and AID::cdk-1 strains to identify genes that are differentially expressed in animals with mononucleated intestines (Supplemental Figure 3F)."

12) For the RNAseq data:

- The statement "possibly growth-delayed or sterile outlier animals were removed from the dataset, based on spermatogenesis and oogenesis-related gene expression" is not explained. The authors provided reference does not seem to explain directly how they identified transcriptomes from growth delayed or sterile outliers.

We have added a more detailed description of how we determined outlier animals in the methods section of the revised version:

Page 23 and 24, lines 544-552:
"Additionally, possibly growth-delayed or sterile outlier animals were removed from the dataset by analysing the expression of spermatogenesis and oogenesis genes as described by Perez and colleagues (33). For this, genes associated with GO terms for oogenesis (n = 50 genes) and spermatogenesis (n = 57 genes) were used to identify outlier animals, and normalized read counts were summed per animal and plotted to identify outlier animals. Presence of outlier animals with

very low levels of oogenesis and spermatogenesis gene expression was found to be independent of batch, condition or genetic background."

- There is a lot of overlap in the lipid transport numbers for the time points the authors identified as significantly different between mono and bi-nucleated worms. Is the phenotype they observed earlier for fitness not very consistent in the samples? Is the phenotype only happening in a certain percentage of worms?

We indeed find a large variability in lipid transport number between embryos/animals (Figure 4F,G), and also in reproductive fitness (Figure 5). Nonetheless, we have performed multiple replicates of the experiments and the phenotypes that we observe are reproducible and consistent. We do not think that the phenotype only happens in a certain percentage of worms, as we do not see clear subpopulations in our data. This is also supported by our recently performed smFISH analysis of *vit-2* mRNA expression in animals with binucleated versus mononucleated intestines (see our response to reviewer #1, point 1): here again we find a large variability in the number of *vit-2* mRNAs per cell, with on average lower amounts of *vit-2* mRNAs in mononucleated cells compared to binucleated cells.

- The DEGs considered were said to be the top 25% regarding absolute log2(foldchange). We think it would be useful to state the minimum log2 fold increase of the list of genes in the 25% top genes at least in the methods and analysis.

We have added this information in the methods section:

Page 24, line 555-558:
"Genes with an adjusted P value below 0.05, and a log2 fold change of gene expression within the top 25% (corresponding to an absolute log2 fold change above 0.514 in AID::knl-1 animals and 0.162 in AID::cdk-1 animals) were considered to be significantly and substantially differentially expressed."

- The authors state: "and only intestinally-expressed genes that were found at least once in every sample were included for downstream analysis (Supplemental Figure 3)". It seems inaccurate when the authors refer to "intestinally-expressed" genes or to the differences in intestinal gene expression of the intestine. The authors did not do tissue-specific RNAseq and thus they should clearly define how they define to be intestine gene expression. Did they use the lists of intestinal genes from the Murphy's lab tissue-specific transcription profiles?

To define intestinally expressed genes, we made use of published transcriptome analyses. In the revised version, we included this information and references to the datasets that we used:

Page 23, line 544-546:
"Further filtering of genes was performed to include only genes for which intestinal expression has been annotated in previous analyses of intestinal gene expression (56-59)."

Review COMMONS

13) In their statement: "Importantly, overexpression of CEH-60 and UNC-62 resulted in similar vit-2 expression levels between mononucleated and binucleated intestines, consistent with an impaired activation by these transcription factors in mononucleated cells. Taken together, these results indicate that binucleation of the intestine is needed to facilitate rapid transcriptional upregulation of gene expression at the L4-to-adult transition, which is important for proper intestinal cell function." To assert this the authors should probably show that overexpression of CEH-60 and UNC-62 also resulted in at least partial rescue of the phenotype they observed by repeating the fitness assay with this system?

As discussed in response to reviewer #1 point 2 and reviewer #2 point 2, we have found that overexpression of CEH-60 and UNC-62 can rescue the lipid loading and brood size defects in the progeny of animals with mononucleated intestines, further supporting our hypothesis that intestinal binucleation is important for proper intestinal cell function. We have include this data in the revised version, see Figure 7C,D and page 15 and 16, line 344-364.

Reviewer #3 (Significance (Required)):

Significance:

The work done in this manuscript is very interesting, original, and contributes to our understanding of the differences between cells that are mononucleate and multinucleate polyploidy. This is of interest to the field of polyploidy and developmental biology. Polyploidization of specific cells is part of normal development and it is not clear why some cell types remain mononucleate and others are polynucleate. This manuscript is one of the first glimpses into this question and is of interest to scientists in the field of cell cycle and cell division (cell biology), normal development (developmental biology), wound healing, and cancer development (health/disease and medicine).

Our expertise is in the field of developmental and cell biology in the context of cell cycle regulation and division. We are particularly interested in understanding the effects of aneuploidy and polyploidy in normal development and disease. For our studies, we combine classical and molecular genetics with cell biological imaging techniques. Recently we have also done RNAseq analysis.
