# Supplementary material for: Endomitosis controls tissue-specific gene expression during development
Source: PLoS Biol. 2022 May 24;20(5):e3001597. doi: 10.1371/journal.pbio.3001597 (PMC9129049; doi:10.1371/journal.pbio.3001597)
Supplement: Attachment — Submitted filename: Response to Reviewers.docx [file pbio.3001597.s019.docx]

Response to Reviewers

We thank the reviewers for their comments and suggestions. Our responses are shown in blue.

**Reviewer #1**: Authors have addressed all my original concerns and should be commended on their rigorous study to elucidate the physiology of binucleation in the C.elegans intestinal cells.  The manuscript reads well and clearly demonstrates the transcriptional adaption of binucleation and its impact of worm fecundity.   This is the first study to date to clearly show in vivo that a cell with multiple nuclei enhances its gene expression compared to a mononucleated cell with equal ploidy.

**Reviewer #2**: In our original review for van Rijnberk et al, we expressed enthusiasm for the development of methods to perturb endomitosis in C. elegans and the potential importance of binucleation in driving the rapid expression of vitellogenins in intestine cells. In this new version, the authors have addressed the major concerns regarding the link between vitellogenin expression and the different phenotypes observed. While the observed defects in brood size and gene expression are relatively mild, they nonetheless appear to be reproducible.

Interestingly, the authors' data suggest that binucleation is critical for driving rapid gene expression at the larva to adult transition (Fig. 6). To test this idea, the authors developed an assay consisting of a heat-shock promoter driving the expression of GFP. While in controls, GFP is rapidly expressed upon heat-shock, in cells with no endomitosis GFP expression is delayed. These experiments were performed using a line that expressed auxin-inducible KNL-1 degradation to prevent binucleation, but whether the effects observed are a consequence of loss of binucleation or an indirect effect of loss of KNL-1 in chromosome segregation is currently unclear. Considering the importance of this experiment to the overall conclusions of this study, it would be useful to test this idea using a different perturbation that blocks binucleation, such as auxin-inducible CDK-1 depletion. Alternatively, the authors should explain the reason why they chose to use the AID::KNL-1 line for these experiments and not the AID::CDK-1 one.

The reason that we performed the experiment with the heat-shock promoter driving GFP expression in the *AID::knl-1* strain is because we are unable to generate transcriptional reporters in the *AID::cdk-1* strain background, because the animals become too sick to maintain. So unfortunately, we are unable to perform the heat-shock experiments with the *AID::cdk-1* line. To address this limitation, we have added these sentences to the Discussion:

*“Interestingly, when we compared the transcriptional activation of a heat-shock reporter driving GFP expression in animals with either binucleated or mononucleated intestinal cells, we found that mononucleated cells display a delay in the accumulation of both gfp mRNA and protein. These results suggest that binucleation is important for rapid upregulation of gene expression in general, likely by promoting transcriptional activity, and perhaps also by increasing protein translation, which remains to be tested. Of note, the transcriptional activation experiments were only performed in the AID::knl-1 strain due to technical limitations of the weakly hypomorphic AID::cdk-1 strain, thus we cannot rule out that the transcriptional defects that we observe are specific to the depletion of KNL-1.”*

Minor comment: There is an error in the labelling of Fig. 7a.

Thank you for pointing this out, we have corrected the mistake.

Reviewer #3: The authors of the manuscript number PBIOLOGY-D-21-03267R1 entitled "Endomitosis controls tissue-specific gene expression during development" have addressed adequately all my previous comments from their first submission. I have a few of comments/questions.

1. The authors propose that CEH-60 and UNC-62 are limiting VIT-2 in mononucleated intestinal cells. Are they expressed differentially in mononucleated cells or are they suggesting they are translated at lower level in these cells?

We do not see a significant difference in the expression levels of *unc-62* or *ceh-60* in mononucleated cells. It is possible that there is a difference in their translation, or alternatively, in their localization to the nucleus. We have added an extra paragraph in the Discussion to speculate about this:

*“In future work, it will be interesting to understand how CEH-60 and UNC-62 could be acting differently in mononucleated or binucleated cells. From our RNA sequencing experiments, we find that unc-62 and ceh-60 are not differentially expressed between mononucleated or binucleated cells, suggesting that differences in CEH-60/UNC-62 activities arise post-transcriptionally. Interestingly, in mononucleated cells we also find reduced expression of the ribosomal subunit rpl-11.2 and the transcriptional activator ets-4, which functions in a variety of different tissues and contexts and also controls the expression of vit-2, vit-3, vit-4, vit-5 and ceh-60 (49). Thus, it is possible that lower levels of rpl-11.2 or ets-4 are leading to a global reduction in protein translation and/or RNA polymerase II-specific transcription in mononucleated cells. Another possibility is that the nuclear accumulation of transcription factors such as UNC-62 and CEH-60 is less efficient in mononucleated cells, resulting in reduced transcription. Interestingly, when we compared the transcriptional activation of a heat-shock reporter driving GFP expression in animals with either binucleated or mononucleated intestinal cells, we found that mononucleated cells display a delay in the accumulation of both gfp mRNA and protein. These results suggest that binucleation is important for rapid upregulation of gene expression in general, likely by promoting transcriptional activity, and perhaps also by increasing protein translation, which remains to be tested. Of note, the transcriptional activation experiments were only performed in the AID::knl-1 strain due to technical limitations of the weakly hypomorphic AID::cdk-1 strain, thus we cannot rule out that the transcriptional defects that we observe are specific to the depletion of KNL-1.”*

2. Other genes downregulated in the intestine include a ribosomal protein (rpl-11.2), and a transcription factor (ets-4) predicted to enable DNA-binding transcription factor activity, RNA polymerase-specific (from WormBase). Lower levels of these two transcripts would be expected to downregulate translation globally and/or RNA polymerase II-specific transcription. How does this possibility reconcile with your findings? Do the authors think it should be addressed in the discussion?

Importantly, the authors do not mention in the discussion or elsewhere that ets-4 transcription factor positively regulates vit-2, vit-3, vit-4 and vit-5, as well as ceh-60. Nor that mutations in ets-4 result in reduced gene expression levels.

These are very interesting hypotheses that could help explain our findings. We have addressed these points in the revised version of the Discussion (see our response to point 1).

3. This is just a comment. It is a bit surprising that overexpression of CEH-60 or UNC-62 completely rescue the phenotypes of animals with mononucleated intestines, given other downregulated genes (spp-5, cpl-1) also affect brood size and/or affect fat content/processing. Should this be a concern or "explained" why might be so?

This is a very good point – one explanation could be that *spp-5* and *cpl-1* are themselves also targets of CEH-60 and UNC-62: ChIP-seq analysis of UNC-62 shows that it binds to the promoters of both *spp-5* and *cpl-1* (data from Dowen, 2019, also on Wormbase), and also to the promoters of *asp-6*, *ets-4*, *cpr-4*, *ZK1320.3* (which are also downregulated in mononucleated intestinal cells) so we think that this is an explanation why overexpression of CEH-60 and UNC-62 is so efficiently rescuing the brood size and lipid loading phenotypes. We have added a paragraph in the Discussion to explain this:

*“Two transcription factors, CEH-60 and UNC-62, have been previously shown to drive the large-scale gene expression changes that occur at the transition from larval development to adulthood (39). Depletion of UNC-62 or CEH-60 decreases the expression of vitellogenins in the intestine, leading to reduced fat and lipid levels in embryos and reduced fitness of the progeny (36, 39, 40, 48). Thus, it is possible that reduced transcriptional activity of UNC-62 or CEH-60 underlies the fitness defects that we observe in the progeny of animals with mononucleated intestines. Consistent with this hypothesis, we find that overexpression of CEH-60 and UNC-62 is sufficient to rescue the reproductive fitness and oocyte lipid loading defects that we observe upon inhibition of endomitosis. These results suggest that impaired CEH-60/UNC-62 activity underlies the defects of animals with mononucleated intestinal cells. Interestingly, in addition to regulating vitellogenin gene expression, UNC-62 also binds the promoters of asp-6, ets-4, cpl-1, cpr-4, spp-5 and ZK1320.3 (39), six other genes that we found to be downregulated in animals with mononucleated intestinal cells. Depletion of ets-4, cpl-1 and spp-5 has been shown to give rise to defects in reproductive fitness (49-51), thus it is possible that increasing their levels by overexpression of CEH-60 and UNC-62 is contributing to the rescue in brood size and lipid loading in the progeny of animals with mononucleated intestinal cells.”*
